# Prevalence of depression, anxiety and post-traumatic stress disorder in health care workers during the COVID-19 pandemic: A systematic review and meta-analysis

**DOI:** 10.1371/journal.pone.0246454

**Published:** 2021-03-10

**Authors:** Yufei Li, Nathaniel Scherer, Lambert Felix, Hannah Kuper

**Affiliations:** International Centre for Evidence in Disability, London School of Hygiene & Tropical Medicine, London, United Kingdom; Universitat Wien, AUSTRIA

## Abstract

**Objective:**

The COVID-19 pandemic has placed health care workers under psychological stress. Previous reviews show a high prevalence of mental disorders among health care workers, but these need updating and inclusion of studies written in Chinese. The aim of this systematic review and meta-analysis was to provide updated prevalence estimates for depression, anxiety and post-traumatic stress disorder (PTSD) among health care workers during the COVID-19 pandemic, benefitting from the inclusion of studies published in Chinese.

**Methods:**

Systematic search of EMBASE, MEDLINE, PsycINFO, Global Health, Web of Science, CINAHL, Google Scholar and the Chinese databases SinoMed, WanfangMed, CNKI and CQVIP, for studies conducted between December 2019 and August 2020 on the prevalence of depression, anxiety and PTSD in health care workers during the COVID-19 pandemic. Studies published in both English and Chinese were included.

**Results:**

Data on the prevalence of moderate depression, anxiety and PTSD was pooled across 65 studies involving 97,333 health care workers across 21 countries. The pooled prevalence of depression was 21.7% (95% CI, 18.3%-25.2%), of anxiety 22.1% (95% CI, 18.2%-26.3%), and of PTSD 21.5% (95% CI, 10.5%-34.9%). Prevalence estimates are also provided for a mild classification of each disorder. Pooled prevalence estimates of depression and anxiety were highest in studies conducted in the Middle-East (34.6%; 28.9%). Subgroup and meta-regression analyses were conducted across covariates, including sampling method and outcome measure.

**Conclusions:**

This systematic review and meta-analysis has identified a high prevalence of moderate depression, anxiety and PTSD among health care workers during the COVID-19 pandemic. Appropriate support is urgently needed. The response would benefit from additional research on which interventions are effective at mitigating these risks.

## Introduction

In December 2019, China experienced the first outbreak of severe acute respiratory syndrome coronavirus 2 (SARS-CoV-2), a highly infectious virus causing the coronavirus disease 2019 (COVID-19). In March 2020, the World Health Organization (WHO) declared this outbreak a global pandemic [1]. As of 21 December 2020, there have been over 76 million documented cases of COVID-19, and over 1.6 million deaths [2].

Coronaviruses are a family of viruses that typically cause serious and sometimes fatal respiratory tract infections, and compared to others, SARS-CoV-2 spreads quickly. The reproductive rate (i.e. number of infected generated by one infected individual, on average) for SARS-CoV-2 is estimated at 2.5, compared with 0.9 for the Middle-East respiratory syndrome coronavirus (MERS-CoV), and 1.5 for the influenza pandemic of 2009 [3]. With SARS-CoV-2 spreading rapidly, health systems across the globe have faced unprecedented challenges in resourcing a health care response [4]. Health care workers have reported inadequate training on infection prevention and control, and there are widespread shortages of personal protective equipment (PPE). These challenges resulted in high rates of COVID-19 among health care workers, especially in the early stages of the pandemic [5–7]. Fears for personal safety, high workload (particularly for those treating infected patients) and limited support may have contributed to fatigue, burnout and stress among health care workers [6]. Although separate constructs, burnout and stress are associated with co-morbid and future psychological outcomes, including common mental disorders, such as depression and anxiety, through various social and biological mechanisms [8–12].

Evidence from previous viral epidemics and initial findings from the COVID-19 pandemic highlight the psychological impact on health care workers [13–15]. The estimated prevalence of depression and anxiety among health care workers was 25% (95% CI, 17%-33%) and 26% (95% CI, 18%-34%), respectively, in a recent systematic review of 19 studies focussed on COVID-19 [16]. In another rapid systematic review, including 29 studies, the median prevalence of anxiety was 24%, and of depression 21% [13]. Comparing these estimates with those from the WHO on common mental disorders among the global population, at 4.4% for depression and 3.6% for anxiety disorders (including PTSD), highlights the substantial impact of the COVID-19 pandemic on the psychological wellbeing of health care workers [17].

Reliable, comprehensive estimates of mental health disorders among health care workers during the COVID-19 pandemic are needed to inform prevention and treatment initiatives. The existing estimates are important, but they need updating. Furthermore, substantial research has been conducted in China, as they were the first country to see an outbreak of COVID-19 and have an active academic community. However, much of this evidence is published in Chinese and stored on Chinese bibliographic databases, and is missed by existing reviews. As noted by Xiang et al., it is imperative that this language barrier does not impede dissemination of findings that will benefit of health professionals and policy makers during this pandemic [18].

In this systematic review and meta-analysis, we aimed to provide updated estimates of the prevalence of depression, anxiety and post-traumatic stress disorder (PTSD) among health care workers during the COVID-19 pandemic, including research published in Chinese.

## Methods

This systematic review was conducted in accordance with the Preferred Reporting Items for Systematic Reviews and Meta-Analysis (PRISMA) guidelines [19]. The protocol for the review was registered in the PROSPERO International Prospective Register of Systematic Reviews in May 2020 (ID: CRD42020187314).

### Search strategy

Articles were retrieved through a systematic search of EMBASE, MEDLINE, PsycINFO, Global Health, Web of Science, Google Scholar and CINAHL, as well as the Chinese databases SinoMed, WanfangMed, CNKI and CQVIP. The reference lists of included studies were searched for additional articles and previous meta-analyses were explored for studies not identified in our search.

Search terms combined items on mental health (depression, anxiety, PTSD), health care workers and COVID-19 (S1 Appendix).

### Inclusion and exclusion criteria

Studies were included if they met the following criteria: (1) published in English or Chinese since the outbreak of COVID-19 in December 2019; (2) report on depression, anxiety or PTSD among health care workers (both clinical and support) in a country affected by COVID-19; (3) used an established assessment of depression, anxiety or PTSD, through either a self-report screening tool or diagnostic interview; (4) provided sufficient information to calculate prevalence of depression, anxiety or PTSD among health care workers (e.g. percentage or sample size and number). We excluded qualitative studies, study protocols and review articles. We did not limit our inclusion to peer-reviewed articles only, and included research letters, briefs and academic preprints stored on servers such as bioRxiv and medRxiv.

### Study selection

The author YL screened all titles and abstracts against the selection criteria, of which 10% were independently screened by author HK. There was 97% agreement between reviewers. When the relevance of the title and abstract was unclear, reviewers consulted the full text. Full texts of selected studies were reviewed by YL and HK, and disagreements on inclusion were discussed until a consensus was reached.

### Data extraction

The following data was extracted from included studies, with use of a standardised form: (1) Study details: country, setting, study design, sampling technique, sample size, response rate; (2) Participant characteristics: gender, age, occupation, proportion of health care workers in direct contact with patients infected with COVID-19; (3) Outcome: diagnostic method or screening tool used, reported prevalence of depression, anxiety and PTSD, prevalence estimates at different thresholds of symptom severity. Data was extracted by author YL and reviewed by NS.

### Quality assessment

All included studies were cross-sectional, and were assessed for risk of bias using the tool from Agarwal et al. ‘Risk of Bias in Cross-Sectional Surveys of Attitudes and Practices’, developed as based on existing tools and response options for observational studies, and used widely [20]. Through this tool, risk of bias was assessed across five domains: (1) Is the source population representative of the population of interest? (2) Is the response rate adequate? (3) Is there little missing data? (4) Is the survey clinically sensible? (5) Is there any evidence for the reliability and validity of the survey instrument?

Each item is rated on a four-point scale from “definitely yes” (low risk of bias) to “definitely no” (high risk of bias). The instrument includes examples of study design that would lead to low risk of bias, higher risk of bias (“probably yes” or “probably no”) and high risk of bias.

### Data analysis

Many studies used more than one cut-off score to report the prevalence of depression, anxiety and PTSD. For the purposes of this study, and for ease of interpretation, we calculated pooled prevalence estimates at two severity levels, classifying them as follows:

- Moderate depression, anxiety or PTSD: the prevalence of health care workers scoring at or above the cut-off for moderate symptomology, or the cut-off defined by the author to be clinically relevant
- Mild depression, anxiety or PTSD: the prevalence of health care workers scoring at or above the cut-off for mild symptomology, or the cut-off defined by the author to be clinically relevant

Pooled prevalence estimates of depression, anxiety and PTSD were calculated by pooling estimates from each study [21]. The Freeman-Tukey double arcsine transformation was applied to stabilise variance [22]. Pooled estimates were calculated using a random-effects model (DerSimonian and Laird method) to account for expected between-study heterogeneity arising from variations in study characteristics, such as country setting, sampling method and mental health screening tool used [23]. Heterogeneity across analyses was assessed using the *I*^*2*^ statistic; a value of 25%, 50% and 75% represents low, medium and high heterogeneity, respectively [24]. Risk of publication bias was assessed through visual inspection of Begg’s funnel plot and Egger’s test [25, 26].

Subgroup analyses were conducted across study characteristics; geographic region, sample size, sampling method, publication status (peer-reviewed vs preprint), proportion of female participations (≤ 50% <), proportion of participants in contact with patients infected with COVID-19 (≤ 50% <) and mental health screening tool. For this review, sampling method was categorised as either random (e.g. simple random sampling, cluster sampling) or non-random (e.g. convenience sampling, voluntary response/self-selection). In each subgroup analysis, *Z*-tests were used to calculate statistically significant inter-group differences in estimated prevalence. To further explore sources of heterogeneity, we conducted random-effects univariate meta-regression analyses for all variables [27]. Covariates significantly associated with heterogeneity were included in a multi-variate meta-regression model. From this model, we calculated the *R*^*2*^ index, in order to quantify the proportion of variance explained by the included covariates. Across analyses, *p*<0.05 indicated statistical significance.

All analyses were conducted using Stata version 16.1., using the metaprop and metareg commands.

## Results

**Fig 1:**
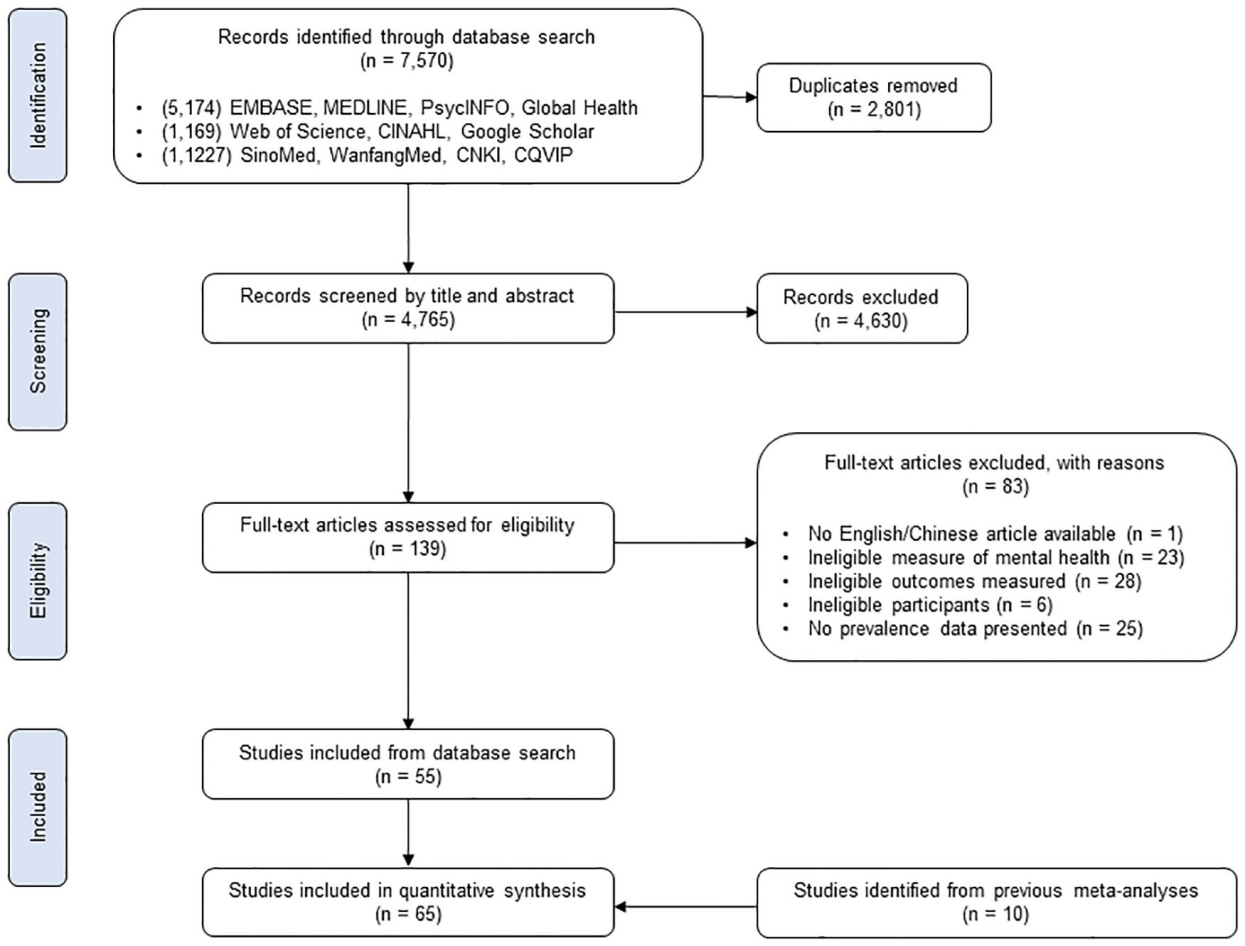
PRISMA flowchart.

The literature search identified 7,570 articles published between 1^st^ December 2019 and 1^st^ August 2020, from which 2,801 duplicates were removed (Fig 1). After screening title and abstracts, 139 articles were eligible for full-text assessment, of which 55 were included [28–82]. An additional 10 articles were extracted from previous meta-analyses, which were not retrieved from the database search [83–92]. In total, 65 studies were included in the analysis. All articles were published in 2020.

### Study characteristics

97,333 health care workers across 21 countries participated in the 65 included studies (S2 Appendix). 46 of these studies were conducted in East Asia, seven in the Middle-East, five in Europe, three in South Asia, one in South America, two in North America and one in West Africa. 43 were conducted in China.

All included studies were cross-sectional in design. Five studies adopted random sampling techniques, whilst the other 60 used non-random methods (for example, self-selection through an online survey, or purposeful sampling). Studies with minimal information on sampling technique were deemed non-random.

70% of participants were female, when identified in 59 studies reporting gender demographics (involving 85,812 health care workers). 45% of participants across all studies were nurses, 27% were doctors, 11% were other medical workers (e.g. technicians, allied health professionals, pharmacists), 1% were administration and support staff, and 17% were health care workers with an undefined occupation. When reported (in 30 studies involving 37,983 participants) 37% of health care workers were in direct contact with patients infected with COVID-19.

All studies used valid self-report mental health screening tools designed to identify the presence of symptoms of common mental disorders. None of the included studies estimated prevalence via clinical diagnostic interview.

Prevalence data for each study is displayed in S3 Appendix, along with additional information on the screening tool and cut-off scores used to define symptom severity. The quality assessment, identifying risk of bias across five domains, is available in S4 Appendix.

### Prevalence of depression

**Fig 2:**
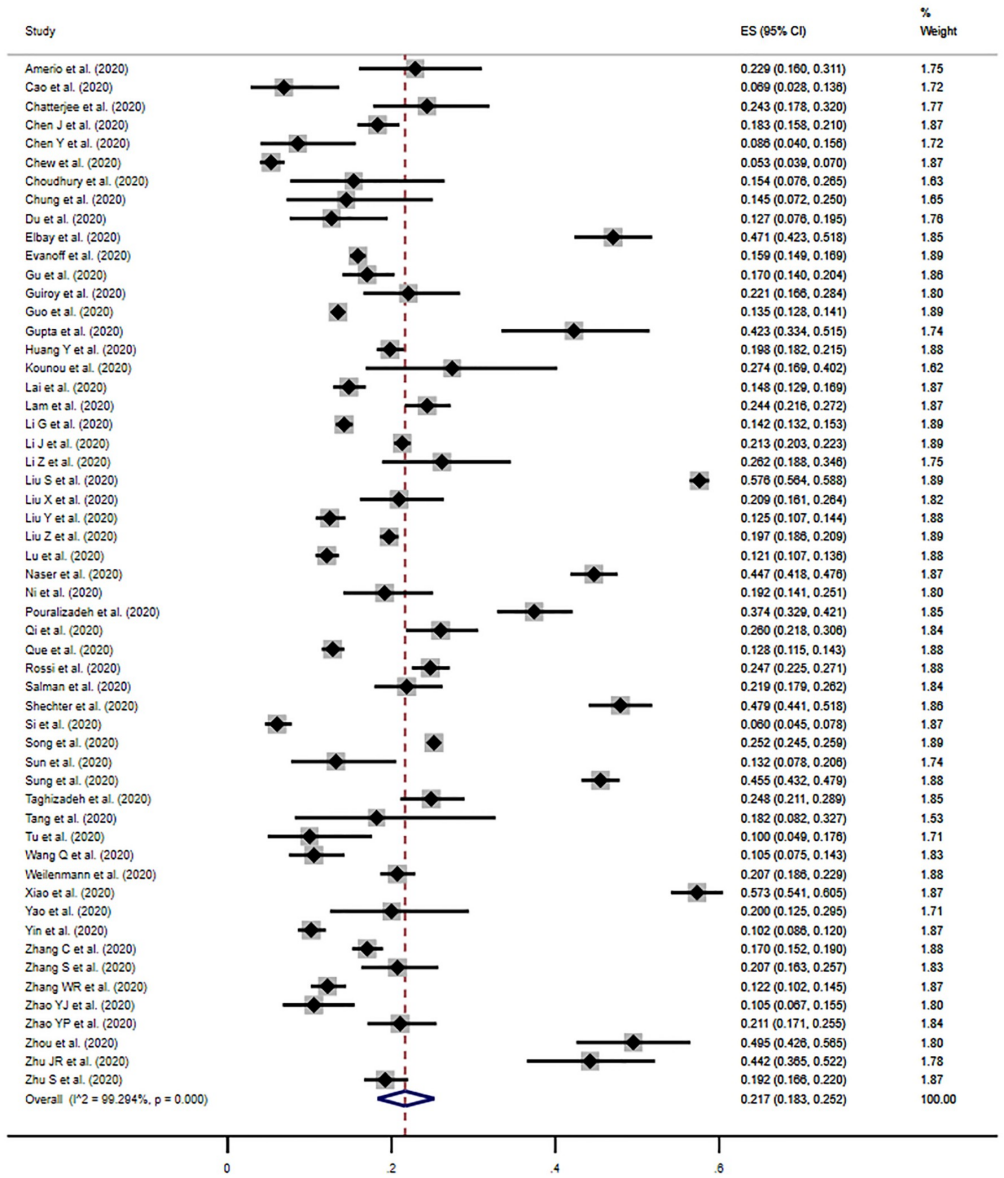
Meta-analysis and pooled estimate of moderate depression in health care workers during the COVID-19 pandemic.

The estimated pooled prevalence of moderate depression was 21.7% (95% CI, 18.3%-25.2%) across 55 studies, when defining depression as a score at or above the cut-off for moderate symptomology, or the cut-off deemed by the author to be clinically relevant (Fig 2). Individual study estimates ranged from 5.3% to 57.6% and there was evidence of high between-study heterogeneity (*I*^*2*^ = 99.3%, *p*<0.001). Although Begg’s funnel plot appeared slightly asymmetric, suggesting marginal bias, Egger’s test provided no evidence of publication bias (*p* = 0.90) (S5A Appendix).

The pooled estimate of mild depression was 36.1% (95% CI, 31.3%-41.0%) when defining the presence of depressive symptoms as a score at or above the cut-off for mild symptomology, or that noted by the author to be clinically relevant (S5B Appendix).

#### Subgroup analysis and meta-regression: Depression

Prevalence estimates of moderate depression were compared between region: East Asia, South Asia, the Middle-East, Europe, North America, West Africa and South America (Table 1). The estimates of these regions significantly differed (*p* = 0.001). Pooled estimates were highest for studies conducted in the Middle-East (34.6%; 95% CI, 25.1%-44.9%), although relatively wide confidence intervals were present. Pooled estimates were lowest in North America (18.7%; 95% CI, 17.8%-19.7%) and East Asia (19.1%; 95% CI, 15.2%-23.4%). Pooling the estimates of the 37 studies from China only did not result in a substantially different estimate to that of all studies from East Asia.

**Table 1:** Subgroup analyses for studies on depression.

Subgroup analysis		No. of studies	Prevalence % (95% CI)	*I*^*2*^	Between-group difference
Region	East Asia	39	19.1 (15.2–23.4)	99.4%	
	Middle-East	5	34.6 (25.1–44.9)	96.6%	
	Europe	4	22.0 (18.9–25.3)	64.3%	
	South Asia	3	28.8 (18.1–40.8)	-^b^	
	North America	2^a^	18.7 (17.8–19.7)	-	
					*p* = 0.001
Sampling method	Non-random	51	22.1 (18.6–25.9)	99.3%	
	Random	4	15.7 (10.7–21.3)	76.0%	
					*p* = 0.06^c^
Screening tool	PHQ-9	28	21.9 (16.2–28.2)	99.4%	
	SDS	7	20.2 (15.5–25.3)	96.9%	
	DASS-21	6	18.7 (9.6–30.0)	98.8%	
	PHQ-2	3	25.1 (5.8–52.1)	-	
	HADS	3	29.2 (6.3–60.2)	-	
	PHQ-4	2	20.8 (17.5–24.3)	-	
	CES-D	2	24.4 (23.8–25.1)	-	
					*p*<0.001

Abbreviation: CES-D = Center for Epidemiologic Studies Depression Scale; DASS = Depression Anxiety Stress Scales; HADS = Hospital Anxiety and Depression Scale; PHQ = Patient Health Questionnaire; SDS = Zung’s Self-Rating Depression Scale.

^a^Only subgroups with two or more included studies are presented.

^b^I^2^ statistic provided for subgroups with four or more included studies.

^c^Borderline significance.

The pooled prevalence estimate of studies using random sampling techniques (15.7%; 95% CI, 10.7%-21.3%) was lower than those using non-random (22.1%; 95% CI, 18.6%-25.9%), although evidence of differential estimates was weak (*p* = 0.06).

28 of the included studies used the PHQ-9 to screen for depressive symptoms, and when estimates were pooled, these studies yielded a prevalence of 21.9% (95% CI, 16.2%-28.2%). The highest pooled prevalence estimate was calculated across the three studies using the HADS (29.2%; 95% CI, 16.3%-60.2%), with the lowest estimate from the six studies using the DASS-21 (18.7%; 95% CI 9.6%-30.0%), although it is worth noting the wide and overlapping confidence intervals, suggesting imprecise estimates. The subgroup analysis suggested evidence of differential prevalence estimates between screening tools (*p*<0.001).

There was no evidence of differential prevalence estimates across other subgroups: sample size (*p* = 0.81); publication status (*p* = 0.30); the proportion of female participants (*p* = 0.91); and the proportion of participants in contact with infected patients (*p* = 0.92). Moreover, none of the covariates included in the meta-regression model explained the presence of heterogeneity.

### Prevalence of anxiety

**Fig 3:**
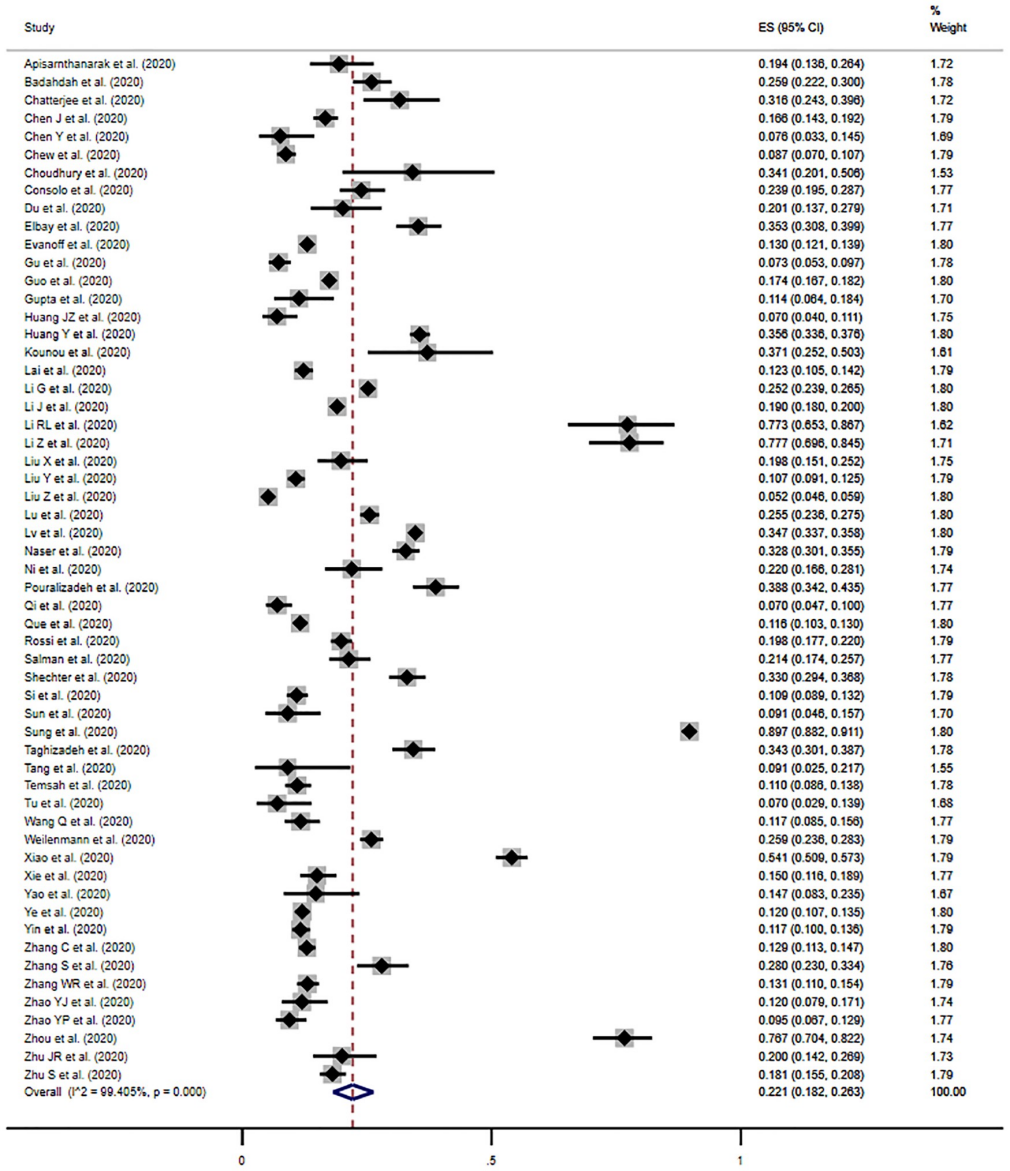
Meta-analysis and pooled estimate of moderate anxiety in health care workers during the COVID-19 pandemic.

The pooled prevalence of moderate anxiety was 22.1% (95% CI, 18.2%-26.3%) across 57 studies, when defining anxiety as a score at or above the cut-off for moderate symptomology, or the cut-off noted by the author to be clinically relevant (Fig 3). Individual study estimates ranged from 5.2% to 89.7%, and there was significant evidence of between-study heterogeneity (*I*^*2*^ = 99.4%, *p*<0.001). Although asymmetry in Begg’s funnel plot indicated a likelihood of publication bias, there was no evidence based on Egger’s test (*p* = 0.63) (S6A Appendix).

The prevalence of mild anxiety was estimated at 38.3% (95% CI, 32.6%-44.3%) when defining the presence of anxiety symptoms as the cut-off for mild anxiety, or the cut-off for a clinically relevant score (S6B Appendix).

#### Subgroup analysis and meta-regression: Anxiety

As presented in Table 2, the prevalence estimates of moderate anxiety differed significantly across region (*p*<0.001). The studies from the Middle-East yielded the highest pooled prevalence estimate (28.9%; 95% CI, 21.6%-36.8%), and the lowest was calculated across the studies of North America (14.8%; 95% CI, 13.9%-15.7%). The 37 studies from China yielded a pooled prevalence (19.1%; 95% CI, 15.5%-23.0%) slightly lower than calculated across all studies from East Asia (20.5%; 95% CI, 15.7%-25.8), although the confidence intervals overlap, suggesting similar distribution of estimates.

**Table 2:** Subgroup analyses for studies on anxiety.

Subgroup analysis		No. of studies	Prevalence % (95% CI)	*I*^*2*^	Between-group difference
Region	East Asia	40	20.5 (15.7–25.8)	99.6%	
	Middle-East	7	28.9 (21.6–36.8)	96.4%	
	Europe	4	23.9 (19.6–28.4)	82.9%	
	South Asia	3	21.0 (11.7–31.4)	-^b^	
	North America	2^a^	14.8 (13.9–15.7)	-	
					*p*<0.001
Sampling method	Non-random	52	23.8 (19.7–28.1)	99.4%	
	Random	5	7.9 (4.4–12.3)	94.0%	
					*p*<0.001
Screening tool	GAD-7	29	20.8 (17.2–24.7)	98.7%	
	SAS	9	10.1 (5.6–15.6)	98.6%	
	DASS-21	6	27.0 (16.1–39.4)	98.8%	
	HADS	3	32.0 (10.8–58.1)	-	
	GAD-2	3	22.1 (10.1–37.2)	-	
	PHQ-4	2	24.1 (20.6–27.7)	-	
	HAMA	2	26.8 (25.0–28.8)	-	
					*p*<0.001
Contact with infected patients	>50%	15	25.7 (17.4–34.9)	98.8%	
	≤50%	12	17.4 (14.5–20.4)	96.2%	
					*p* = 0.06^c^

Abbreviation: DASS = Depression Anxiety Stress Scales; GAD = Generalised Anxiety Disorder Assessment; HADS = Hospital Anxiety and Depression Scale; HAMA = Hamilton Anxiety Rating Scale; PHQ = Patient Health Questionnaire; SAS = Zung’s Self-Rating Anxiety Scale

^a^Only subgroups with two or more included studies are presented

^b^I^2^ statistic provided for subgroups with four or more included studies

^c^Borderline significance

The prevalence estimate across studies adopting a random sampling methodology (7.9%; 95% CI, 4.4%-12.3%) was significantly lower (*p*<0.001) than the pooled estimate across studies using non-random methods (23.8%; 95% CI, 19.7%-28.1%), and was 14.2% lower than the overall pooled estimate.

29 studies used the GAD-7, and the pooled prevalence estimate across these studies was 20.8% (95% CI, 17.2%-24.7%). The highest pooled prevalence was calculated from the studies using HADS (32.0%; 95% CI, 10.8%-58.1%), although this group included only three studies and the confidence intervals are wide. Those studies using the SAS yielded the lowest pooled estimate (10.1%; 95% CI, 5.6%-15.6%), although it should be noted that the confidence intervals overlap with those of the HADS. The pooled estimates of these subgroups differed significantly (*p*<0.001).

Data on the proportion of participants in contact with patients infected with COVID-19 was provided in 27 studies only. Studies in which more than 50% of participants were in contact with patients with COVID-19 demonstrated a higher prevalence of anxiety (25.7%; 95% CI, 17.4%-34.9%), compared to studies in which 50% or fewer participants were in contact (17.4%; 14.5%-20.4%), although evidence of this difference was of borderline significance (*p* = 0.06).

Prevalence estimates did not significantly differ based on sample size (*p* = 0.73); publication status (*p* = 0.13); and the proportion of female participants (*p* = 0.25). Based on the univariate meta-regression analyses, there was evidence that the following variables explained between-study heterogeneity: sampling method (*p* = 0.03); screening tool (*p* = 0.05); publication status (*p* = 0.03); and the proportion of participants in contact with infected patients (*p* = 0.04). The subsequent multi-variate meta-regression model suggested that these variables explained approximately 17% of the between-study variance (adjusted *R*^*2*^ = 17.4%).

### Prevalence of PTSD

**Fig 4:**
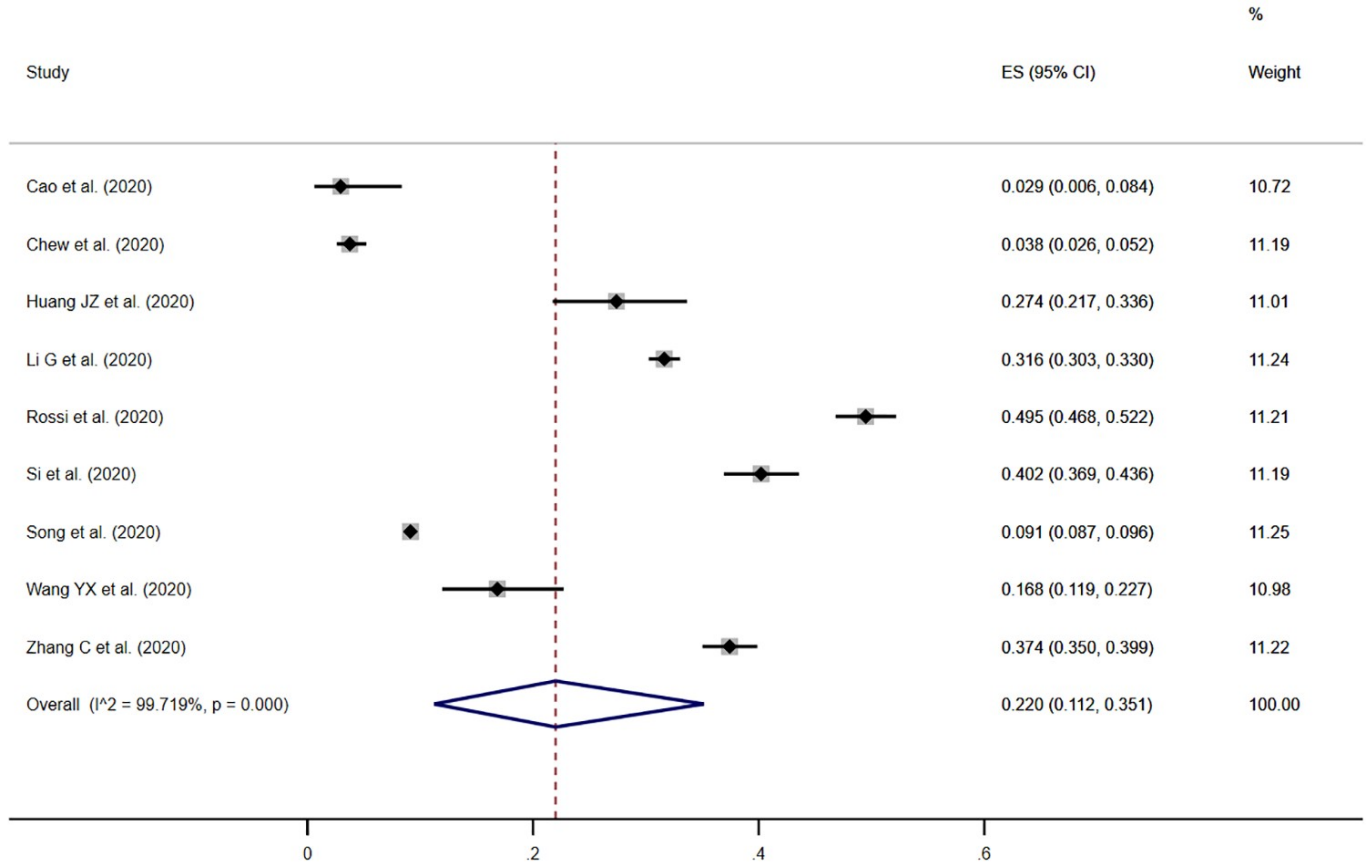
Meta-analysis and pooled estimate of moderate PTSD in health care workers during the COVID-19 pandemic.

Symptoms of PTSD in health care workers was measured in nine studies. The pooled prevalence estimate of moderate PTSD was 21.5% (95% CI, 10.5%-34.9%) when defined as a score at or above the cut-off for moderate symptomology, or the cut-off noted by the author to be clinically relevant (Fig 4). Individual study estimates ranged from 2.9% to 49.5%, and there was evidence of between-study heterogeneity (*I*^*2*^ = 99.7%, *p*<0.001).

Seven of the nine studies reported the prevalence estimate of health care workers scoring at or above a single severity threshold, and we have not presented estimates of mild PTSD. Subgroup analyses and an assessment of publication bias were not undertaken because of the small number of studies (<10) reporting the prevalence of PTSD.

## Discussion

This systematic review and meta-analysis of 65 studies involving 97,333 health care workers across 21 countries demonstrated high prevalence estimates of moderate depression (21.7%), anxiety (22.1%) and PTSD (21.5%) among health care workers during the COVID-19 pandemic, consistent with findings of previous reviews [13, 16].

Most of the studies in this review used non-random sampling methods, which may have led to selection bias and over-estimation of the prevalence of these disorders. This pattern is particularly notable in the prevalence estimates for anxiety, in which studies with random sampling yielded a pooled estimate of 7.9%, 14.2% lower than the summary estimate. Notwithstanding this concern, the estimates for depression, anxiety and PTSD are considerably higher than those expected among the general population in regular times (depression: 4.4%; anxiety, including PTSD: 3.6%), calling attention to the considerable psychological impact of the pandemic on health care workers [17]. The evidence is clear, those with mental disorders are more likely to experience excess morbidity and premature mortality, as well as negative impacts across work, education and community life [93, 94].

When interpreting the pooled prevalence estimates calculated in this review and meta-analysis, it is important to note that the percentage of variability (*I*^*2*^) in the prevalence estimates due to heterogeneity was very high. That said, the *I*^*2*^ is extremely sensitive when a large number of studies are included in meta-analyses, and a high *I*^*2*^ is often inevitable [95]. The *I*^*2*^ may therefore detect only a small amount of heterogeneity, which is not clinically important. Despite this, we explored heterogeneity based on subgroup and meta-regression analyses. Although the subgroup analyses suggested evidence of between-group differences in prevalence estimates across a number of variables (e.g. region, sampling method, screening tool), the meta-regression did not adequately explain the presence of heterogeneity. With regards to the pooled estimate of moderate anxiety, the included variables explained only 17.4% of between-study heterogeneity.

Nevertheless, observations across included studies suggest that health care workers are at risk of common mental disorders during the pandemic. We earlier compared the prevalence estimates of our meta-analysis with those from the WHO among the general population in regular times, to highlight the impact of the pandemic on mental health. With the estimates from the WHO and those presented in this paper generated through different methods, we did not think it appropriate to undertake statistical comparison, and have provided a narrative commentary. We were not able to source global estimates of common mental disorders among health care workers in regular times, but this would be a valuable comparison.

Our subgroup analyses identified a higher prevalence of anxiety in studies with a greater proportion of participants in direct contact with patients infected with COVID-19, although the evidence was weak. This finding is consistent with the association of anxiety and workplace fatigue, burnout and fear for one’s safety, likely higher in workers in direct contact with infected patients [6]. Not all studies provided information on the number of participants in contact with infected patients, and of those that did, none provided disaggregated prevalence data. Providing this information in future studies will aid further evidence syntheses and analyses. Similarly, disaggregated data by other variables (e.g. gender, occupation) would provide opportunity for more detailed analysis, and allow us to better understand the risk factors associated with common mental disorders, needed to inform an appropriate response. We encourage future research to identify risk factors present in different settings.

Although the prevalence estimates were imprecise (wide confidence intervals), it is suggested that health care workers in the Middle-East experience high prevalence of depression and anxiety, perhaps attributed to this region’s relatively high COVID-19 caseload [2]. Although China saw the first outbreak of COVID-19, the reported caseload is substantially lower than reported in other countries and regions, and this may be a factor in the relatively lower pooled estimates calculated for the country, and across the East Asia region. Variation in sampling methods is an alternative explanation. Most of the included literature adopted non-random sampling techniques, increasing the risk of bias in individual studies, and potentially resulting in overestimation of the prevalence of mental health disorders. Each study using random sampling was conducted in China, and this may also explain the lower prevalence estimates pooled across this country and East Asia.

Most studies used online surveys and questionnaires to assess mental health status (examples of non-random self-selection). These would have been necessary during the COVID-19 pandemic, with many countries practicing self-isolation and social distancing measures, making face-to-face assessment challenging and dangerous. Evidence suggests that remote, online screening results in comparable estimates to face-to-face delivery, and these methods, in and of themselves, are not a concern [96, 97]. That being said, their use is associated with non-random sampling methodologies, and consequently increases the chance of selection bias. Future research should use face-to-face survey methodologies, where possible, to reduce the risk of bias.

### Strengths and limitations

The primary strength of this study is its search of international and Chinese databases, and its inclusion of studies published in both English and Chinese. Adhering to PRISMA guidelines and systematic review methods provided methodological rigour.

This study has important limitations to consider. Not all studies were screened by two reviewers at title and abstract, and although evidence suggests that limited dual review processes remain an effective procedure, there may have been eligible studies missed [98, 99]. Additionally, it is possible we missed some relevant literature as only articles in English or Chinese were included, and some databases were not searched (e.g. Scopus). Further, we included preprint articles, not yet peer-reviewed, and results from these studies may change in the future and methodological biases may be present. That being said, results of our subgroup analyses did not indicate differential prevalence estimates between peer-reviewed and preprint papers. The majority of data were derived from studies using different sampling methods, study design, screening tools and diagnostic thresholds, and substantial heterogeneity was seen. We were unable to explain much of this variability in our meta-regression models, as we were restricted with the information on covariates available to us. Although visual inspection of Begg’s funnel plots suggested marginal publication bias, results of Egger’s tests did not; a less subjective methodology and reasonable to rely on, when a meta-analysis includes a relatively large number of studies, as is the case [100]. None of the studies used the gold standard diagnostic interview to identify mental health status, although they each used a validated self-report measure, common methodology in mental health research. Lastly, many studies were conducted with health care workers in a single setting, limiting insight into the generalisability of findings.

## Conclusion

This systematic review and meta-analyses provide the most comprehensive information on the prevalence of depression, anxiety and PTSD among health care workers during the COVID-19 pandemic, to date. Health care workers are at risk of common mental disorders, and the results of this review should inform action in policy and practice, to support the psychological wellbeing of health care workers. Additional research should be conducted into the factors associated with poor mental health, and future prevalence studies must adopt random sampling methods to improve the precision of estimates.

Our findings present a concerning outlook for health care workers, a group continually needed at the forefront of action against COVID-19, and at continued risk of associated psychological stressors. The response from policy makers and service providers must be decisive and swift, addressing mental health concerns in in this group, before long-term health and social impacts are realised. Support initiatives developed during the pandemic can help inform and inspire ideas in service provision across different regions, as the global society combats this pandemic (for example, e-learning to support the psychological wellbeing of health care employees) [101]. There must now be more attention given to generating and assessing the effectiveness of different interventions and initiatives to support the mental health of health care workers during this pandemic.

## Supporting information

S1 Appendix Search strategy. (PDF)

S2 Appendix Study characteristics. (PDF)

S3 Appendix Prevalence data. (PDF)

S4 Appendix Quality assessment. (PDF)

S5 Appendix Supplementary materials: Depression. (PDF)

S6 Appendix Supplementary materials: Anxiety. (PDF)

S7 Appendix PRISMA checklist. (PDF)
