# Supplementary material for: Prevalence of depression, anxiety and post-traumatic stress disorder in health care workers during the COVID-19 pandemic: A systematic review and meta-analysis
Source: PLoS One. 2021 Mar 10;16(3):e0246454. doi: 10.1371/journal.pone.0246454 (PMC7946321; doi:10.1371/journal.pone.0246454)
Supplement: S1 Appendix — (PDF) [file pone.0246454.s001.pdf]

S1 Appendix A. Search terms

Mental health	Health care worker	COVID-19
International database (e.g. MEDLINE)		
"mental health", "psychological", "mood" (burden, condition, problem, disorder, distress, illness, injury)<br><br>"resilience", "wellbeing"<br><br>"post-traumatic stress disorder", "adjustment disorder", "trauma", "depression", "anxiety"	"health care", "hospital", "medical" (worker, professional, personnel, staff, provider)<br><br>"doctor", "physician", "registrar", "consultant", "nurse", "paramedic", "midwife", "surgeon", "general practitioner", "frontline worker", "health care assistant", "allied health professional"<br><br>"intensive care", "critical care", "outpatient"	"SARS-CoV-2", "2019-nCoV", "coronavirus", "novel coronavirus", "Wuhan coronavirus", "COVID-19"
Chinese database (e.g. SinoMed)		
"心理健康"、"心理问题"、"心理障碍"、"心理困扰"、"精神紊乱"、"精神问题"、"精神障碍"、"精神健康"、"心理负担"、"精神负担"、"精神疾病"、"心理疾病"、"压力"、"恐惧"、"情绪"、"心理状态"、"创伤后应激障碍"、"PTSD"、"焦虑"、"焦虑症"、"创伤"、"抑郁症"、"抑郁症状"、"抑郁"	"健康从业者"、"医护人员"、"医疗人员"、"医疗工作者"、"医生"、"护士"、"产科"、"重症监护"、"护理人员"、"全科医生"、"门诊"、"急诊"、"医院"	"新冠"、"新冠肺炎"、"新冠病毒"、"新冠疫情"、"新型冠状病毒"、"COVID-19"、"2019冠状病毒"、"严重急性呼吸系统综合征冠状病毒2"

S1 Appendix B. Example search strategy: MEDLINE

**1**	"(Mental health adj3 (burden* or condition* or problem* or distress* or disorder* or illness* or injur*))" OR "(Psychological adj3 (burden* or condition* or problem* or distress* or disorder* or illness* or injur*))" OR "(MOOD adj3 (burden* or condition* or problem* or distress* or disorder* or illness* or injur*))" OR "Depress*" OR "Anxiety" OR "Anxiety disorder*" OR "Affective symptom*" OR "Post?traumatic stress*" OR "PTSD" OR "Adjustment disorder*" OR "Secondary traumatic stress*" OR "Neuros*" OR "Panic" OR "Fear" OR "Breakdown" OR "Wellbeing" OR "Resilience".mp. [mp=title, abstract, original title, name of substance word, subject heading word, floating sub-heading word, keyword heading word, protocol supplementary concept word, rare disease supplementary concept word, unique identifier, synonyms]
**2**	"(Health care adj (worker* or professional* or personnel or staff or employee* or provider* or physician* or assistant))" OR "(Hospital adj (worker* or professional* or personnel or staff or employee* or provider* or physician* or assistant))" OR "(Medical adj (worker* or professional* or personnel or staff or employee* or provider* or physician* or assistant))" OR "(Nursing adj (staff or personnel))" OR "Doctor*" OR "Registrar*" OR "Consultant*" OR "Nurse*" OR "Paramedic*" OR "Allied health worker*"OR "Midwi*" OR "Surgeon*" OR "General practitioner*" OR "Frontline worker*" OR "(Intensive adj (care or care unit or care nursing))" OR "Emergency department*" OR "Outpatient*" OR "Critical care".mp. [mp=title, abstract, original title, name of substance word, subject heading word, floating sub-heading word, keyword heading word, protocol supplementary concept word, rare disease supplementary concept word, unique identifier, synonyms]
**3**	"SARS-CoV-2" OR "2019-nCoV" OR "New coronavirus" OR "Novel coronavirus" OR "Wuhan coronavirus" OR "COVID-19".mp. [mp=title, abstract, original title, name of substance word, subject heading word, floating sub-heading word, keyword heading word, protocol supplementary concept word, rare disease supplementary concept word, unique identifier, synonyms]
**4**	1 AND 2 AND 3
