# Supplementary material for: Prevalence of depression, anxiety and post-traumatic stress disorder in health care workers during the COVID-19 pandemic: A systematic review and meta-analysis
Source: PLoS One. 2021 Mar 10;16(3):e0246454. doi: 10.1371/journal.pone.0246454 (PMC7946321; doi:10.1371/journal.pone.0246454)
Supplement: S2 Appendix — (PDF) [file pone.0246454.s002.pdf]

S2 Appendix. Selected characteristics of the 65 studies included in this review

Study	Country	Publication status	Sampling method	Sample size	Response rate	Age: mean, SD	Gender: female	Occupation	Direct contact with patients with COVID-19
Amerio et al. (2020)	Italy	Peer-reviewed	Non-random	131	25%	52.3, 12.2	63	General practitioner: 131	N/A
Apisarnthanarak et al. (2020)	Thailand	Peer-reviewed	Non-random	160	N/A	32	95	Physician: 52<br>Nurse: 45<br>Assistant nurse: 16<br>Other: 47	82
Badahdah et al. (2020)	Oman	Peer-reviewed	Non-random	509	N/A	37.7	407	Physician: 194<br>Nurse: 315	144
Cao et al. (2020)	China	Preprint	Non-random	102	97.1%	31.7, 6.8	77	Doctor: 40<br>Nurse: 54<br>Technician: 8	93
Chatterjee et al. (2020)	India	Peer-reviewed	Non-random	152	N/A	42.1, 12.2	33	Doctor (non-specialist): 44<br>Medical and allied professional: 58<br>Surgery and allied professional: 33<br>Administration: 17	N/A
Chen J et al. (2020)	China	Peer-reviewed	Non-random	902	N/A	N/A	619	Doctor: 541<br>Nurse: 311<br>Other: 48	N/A
Chen Y et al. (2020)	China	Peer-reviewed	Non-random	105	84.7%	32.6, 6.5	95	Medical worker: 105	N/A
Chew et al. (2020)	Singapore, India	Peer-reviewed	Non-random	906	N/A	29	583	Physician: 268<br>Nurse: 355<br>Allied health: 96<br>Technician: 40<br>Clerical staff: 56<br>Administration: 39<br>Maintenance: 52	
Choudhury et al. (2020)	UK	Peer-reviewed	Non-random	106	N/A	41	41	Physician: 23<br>Nurse and allied health: 23<br>Administration: 17	47
Chung et al. (2020)	China; Hong Kong	Peer-reviewed	Non-random	69	N/A	N/A	N/A	Doctor: 3<br>Nurse: 24<br>Allied health professional: 6<br>Administration: 23<br>Management: 2<br>Other: 3	N/A

Study	Country	Publication status	Sampling method	Sample size	Response rate	Age: mean, SD	Gender: female	Occupation	Direct contact with patients with COVID-19
Consolo et al. (2020)	Italy	Peer-reviewed	Non-random	356	40.0%	N/A	141	Dentist: 356	N/A
Du et al. (2020)	China	Peer-reviewed	Non-random	134	43.1%	N/A	81	Doctor: 47<br>Nurse: 55<br>Support staff: 32	N/A
Elbay et al. (2020)	Turkey	Peer-reviewed	Non-random	442	N/A	36.1, 8.7	251	Surgical specialist: 75<br>Non-surgical specialist: 344<br>Basic medical sciences: 18	231
Evanoff et al. (2020)	USA	Peer-reviewed	Non-random	5,550	34.4%	N/A	4,374	Faculty: 870<br>Staff: 4,470<br>Post-doctoral scholars: 210	425
Gu et al. (2020)	China	Peer-reviewed	Non-random	564	100%	N/A	N/A	Nurse: 564	564
Guiroy et al. (2020)	Argentina, Brazil, Chile, Mexico	Peer-reviewed	Non-random	204	43.3%	47.8	7	Orthopaedist: 120<br>Neurosurgeon: 84	N/A
Guo et al. (2020)	China	Peer-reviewed	Non-random	11,118	N/A	N/A	8,316	Physician: 3,351<br>Nurse: 5,900<br>Medical student: 757<br>Medical assistant: 464<br>Administration: 450	3,351
Gupta et al. (2020)	India	Peer-reviewed	Non-random	123	N/A	N/A	N/A	Health professional: 123	N/A
Huang JZ et al. (2020)	China	Peer-reviewed	Random	230	93.5%	N/A	187	Doctor: 70<br>Nurse: 160	N/A
Huang Y et al. (2020)	China	Peer-reviewed	Non-random	2,250	N/A	N/A	N/A	Health professional: 2,250	N/A
Kounou et al. (2020)	Togo	Peer-reviewed	Non-random	62	N/A	N/A	35	Medical professional: 62	N/A
Lai et al. (2020)	China	Peer-reviewed	Random	1,257	68.7%	N/A	964	Physician: 493<br>Nurse: 764	522
Lam et al. (2020)	China; Hong Kong	Peer-reviewed	Non-random	932	N/A	N/A	701	Physician: 263<br>Nurse: 580<br>Other: 89	359
Li G et al. (2020)	China	Peer-reviewed	Non-random	4,369	82.2%	N/A	4,369	Doctor: 582<br>Nurse: 3,380<br>Medical technician: 407	N/A
Li J et al. (2020)	China	Preprint	Non-random	4,004	N/A	N/A	2,238	Public health worker: 6,317	N/A

Study	Country	Publication status	Sampling method	Sample size	Response rate	Age: mean, SD	Gender: female	Occupation	Direct contact with patients with COVID-19
Li RL et al. (2020)	China	Peer-reviewed	Non-random	66	97.1%	N/A	51	Nurse: 66	66
Li Z et al. (2020)	China	Peer-reviewed	Random	130	97.7%	36.1, 5.6	121	Nurse: 130	130
Liu S et al. (2020)	China	Peer-reviewed	Non-random	6,588	N/A	N/A	4,370	Medical professional: 2,543<br>Nurse: 3,888<br>Allied health: 150<br>Auxiliary services: 27	N/A
Liu X et al. (2020)	China	Preprint	Non-random	258	N/A	N/A	173	Medical staff: 258	96
Liu Y et al. (2020)	China	Preprint	Non-random	1,315	N/A	37	999	Physician: 512<br>Nurse: 577<br>Technician: 120<br>Hygienist: 106	1,315
Liu Z et al. (2020)	China	Preprint	Non-random	4,679	N/A	35.9, 9	3,849	Doctor: 1,853<br>Nurse: 2,826	1,437
Lu et al. (2020)	China	Peer-reviewed	Non-random	2,042	N/A	N/A	1,785	Medical staff: 2,042	N/A
Lv et al. (2020)	China	Preprint	Non-random	8,028	N/A	N/A	N/A	Medical staff: 8,028	N/A
Naser et al. (2020)	Jordan	Peer-reviewed	Non-random	1,163	N/A	N/A	653	Physician: 560<br>General practitioner: 100<br>Nurse: 151<br>Allied health: 74<br>Pharmacist: 378	617
Ni et al. (2020)	China	Peer-reviewed	Non-random	214	N/A	N/A	148	Doctor: 81<br>Nurse: 108<br>Other: 25	55
Pouralizadeh et al. (2020)	Iran	Peer-reviewed	Non-random	441	N/A	36.3, 8.7	420	Nurse: 441	412
Qi et al. (2020)	China	Peer-reviewed	Non-random	400	100%	N/A	295	Nurse: 400	30
Que et al. (2020)	China	Peer-reviewed	Non-random	2,285	N/A	31.1	1,578	Physician: 860<br>Medical resident: 913<br>Nurse: 208<br>Technician: 179<br>Public health professional: 125	N/A
Rossi et al. (2020)	Italy	Peer-reviewed	Non-random	1,379	49.0%	39	1,064	Physician: 433<br>Nurse: 472<br>General practitioner: 86<br>Health care assistant: 112<br>Other: 275	725

Study	Country	Publication status	Sampling method	Sample size	Response rate	Age: mean, SD	Gender: female	Occupation	Direct contact with patients with COVID-19
Salman et al. (2020)	Pakistan	Preprint	Non-random	398	N/A	28.7	215	Doctor: 205<br>Nurse: 133<br>Pharmacist: 60	N/A
Shechter et al. (2020)	USA	Peer-reviewed	Non-random	657	13.7%	N/A	509	Attending physician: 141<br>Resident or fellow: 141<br>Nurse: 313<br>Advanced practice provider: 48	N/A
Si et al. (2020)	China	Peer-reviewed	Non-random	863	76.0%	N/A	610	Doctor: 377<br>Nurse: 211<br>Other: 275	145
Song et al. (2020)	China	Peer-reviewed	Non-random	14,825	N/A	34	9,536	Physician: 6,093<br>Nurses: 8,732	N/A
Sun et al. (2020)	China	Peer-reviewed	Random	121	97.6%	35.1, 8.9	81	Doctor: 27<br>Nurse: 94	N/A
Sung et al. (2020)	Taiwan	Preprint	Non-random	1,795	N/A	36.7	1,435	Doctor: 357<br>Nurse: 1,064<br>Pharmacist: 20<br>Medical technician: 93<br>Radiation technician: 91	N/A
Taghizadeh et al. (2020)	Iran	Preprint	Non-random	487	59.0%	N/A	N/A	Doctor: 127<br>Nurse: 105<br>Health staff: 229<br>Lab staff: 26	N/A
Tang et al. (2020)	China	Peer-reviewed	Non-random	44	100%	33.6, 6.4	34	Nurse: 441	31
Temsah et al. (2020)	Saudi Arabia	Peer-reviewed	Non-random	582	71.8%	36	437	Senior physician: 56<br>Registrar physician: 52<br>Resident physician: 48<br>Intern: 34<br>Nurse and midwife: 363<br>Auxiliary services: 29	N/A
Tu et al. (2020)	China	Peer-reviewed	Random	100	100%	34.4	100	Nurse: 100	100
Wang Q et al. (2020)	China	Peer-reviewed	Non-random	342	N/A	N/A	314	Nurse: 342	N/A
Wang YX et al. (2020)	China	Peer-reviewed	Non-random	202	95.7%	N/A	177	Nurse: 202	N/A
Weilenmann et al. (2020)	Switzerland	Preprint	Non-random	1,410	N/A	34	934	Physician: 857<br>Nurse: 553	1,103
Xiao et al. (2020)	China	Peer-reviewed	Non-random	958	N/A	N/A	644	Physician: 378<br>Nurse: 359<br>Technician: 119	678

Study	Country	Publication status	Sampling method	Sample size	Response rate	Age: mean, SD	Gender: female	Occupation	Direct contact with patients with COVID-19
Xie et al. (2020)	China	Peer-reviewed	Non-random	394	100%	38.8, 9.7	296	Doctor: 136<br>Nurse: 258	N/A
Yao et al. (2020)	China	Peer-reviewed	Non-random	95	N/A	N/A	82	Physician: 34<br>Nurse: 61	95
Ye et al. (2020)	China	Peer-reviewed	Non-random	2,140	99.6%	N/A	1,644	Nurse: 2,140	N/A
Yin et al. (2020)	China	Peer-reviewed	Non-random	1,266	97.5%	N/A	946	Doctor: 504<br>Nurse: 766	N/A
Zhang C et al. (2020)	China	Peer-reviewed	Non-random	1,563	N/A	N/A	1,293	Doctor: 454<br>Nurse: 984<br>Administration: 30<br>Other: 95	689
Zhang S et al. (2020)	Iran	Peer-review	Non-random	304	N/A	35.1	178	Health worker: 304	N/A
Zhang WR et al. (2020)	China	Peer-review	Non-random	927	N/A	N/A	678	Medical worker: 927<br>Non-medical worker: 1,255	138
Zhao YJ et al. (2020)	China	Peer-review	Non-random	209	99.5%	N/A	130	Chief physician: 2<br>Deputy chief physician: 18<br>Attending physician: 106<br>Resident physician: 52<br>General practitioner: 31	N/A
Zhao YP et al. (2020)	China	Peer-review	Non-random	380	N/A	31.8, 7.3	104	Nurse: 380	18
Zhou et al. (2020)	China	Preprint	Non-random	210	95.4%	30.5, 4.5	105	Doctor: 63<br>Nurse: 147	N/A
Zhu JR et al. (2020)	China	Peer-reviewed	Non-random	165	N/A	34.2, 8.1	137	Doctor: 79<br>Nurse: 86	N/A
Zhu S et al. (2020)	China	Peer-reviewed	Non-random	858	N/A	N/A	597	Frontline staff: 320<br>Non-frontline staff: 539	320

N/A = Information not provided in the article or non-applicable
