# Supplementary material for: Prevalence of depression, anxiety and post-traumatic stress disorder in health care workers during the COVID-19 pandemic: A systematic review and meta-analysis
Source: PLoS One. 2021 Mar 10;16(3):e0246454. doi: 10.1371/journal.pone.0246454 (PMC7946321; doi:10.1371/journal.pone.0246454)
Supplement: S3 Appendix — (PDF) [file pone.0246454.s003.pdf]

S3 Appendix. Prevalence of depression, anxiety and PTSD at different cut-off scores on the screening tools used

Study	Sample size	Screening tool: Depression	Prevalence; Cut-off score: %	Screening tool: Anxiety	Prevalence; Cut-off score: %	Screening tool: PTSD	Prevalence; Cut-off score: %
Amerio et al.	131	PHQ-9	Moderate (≥10): 22.9%	N/A	N/A	N/A	N/A
Apisarnthanarak et al.	160	N/A	N/A	GAD-7	Mild (≥5): 42.5%<br>Moderate (≥10): 19.4%	N/A	N/A
Badahdah et al.	509	N/A	N/A	GAD-7	Mild (≥5): 64.6%<br>Moderate (≥10): 25.9%	N/A	N/A
Cao et al.	102	PHQ-9	Screen positive (≥10): 6.9%	N/A	N/A	IES-R	Screening positive (≥26): 2.9%
Chatterjee et al.	152	DASS-21	Mild (≥10): 34.9%<br>Moderate (≥14): 24.3%	DASS-21	Mild (≥8): 39.5%<br>Moderate (≥10): 31.6%	N/A	N/A
Chen J et al.	902	PHQ-9	Mild (≥5): 48.7%<br>Moderate (≥10): 18.3%	GAD-7	Mild (≥5): 47.0%<br>Moderate (≥10): 16.6%	N/A	N/A
Chen Y et al.	105	SDS	Mild (≥53): 29.5%<br>Moderate (≥63): 8.6%	SAS	Mild (≥50): 18.1%<br>Moderate (≥60): 7.6%	N/A	N/A
Chew et al.	906	DASS-21	Mild (≥9): 10.6%<br>Moderate (≥14): 5.3%	DASS-21	Mild (≥7): 15.7%<br>Moderate (≥10): 8.7%	IES-R	Mild (≥24): 7.4%<br>Moderate (≥33): 3.8%
Choudhury et al.	106	PHQ-9	Mild (≥5): 53.0%<br>Moderate (≥10): 15.0%	GAD-7	Mild (≥5): 61.0%<br>Moderate (≥10): 34.0%	N/A	N/A
Chung et al.	69	PHQ-9	Mild (≥10): 34.8%<br>Moderate (≥15): 14.5%	N/A	N/A	N/A	N/A
Consolo et al.	356	N/A	N/A	GAD-7	Mild (≥5): 57.2%<br>Moderate (≥10): 23.9%	N/A	N/A
Du et al.	134	BDI-II	Screen positive (≥14): 12.7%	BAI	Screening positive (≥8): 20.1%	N/A	N/A
Elbay et al.	442	DASS-21	Mild: 64.7%<br>Moderate: 47.1%	DASS-21	Mild: 51.6%<br>Moderate: 35.2%	N/A	N/A
Evanoff et al.	5,550	DASS-21	Moderate: 15.9%	DASS-21	Moderate: 13.0%	N/A	N/A
Gu et al.	564	SDS	Mild: 37.4%<br>Moderate: 17.0%	SAS	Mild: 30.1%<br>Moderate: 7.3%	N/A	N/A
Guiroy et al.	204	PHQ-9	Moderate (≥10): 22.1%	N/A	N/A	N/A	N/A
Guo et al.	11,118	SDS	Mild (≥50): 31.5%<br>Moderate (≥60): 13.5%	SAS	Mild (≥50): 17.5%<br>Moderate (≥60): 5.0%	N/A	N/A
Gupta et al.	123	PHQ-9	Screen positive (≥5): 42.6%	GAD-7	Moderate (≥10): 11.3%	N/A	N/A
Huang JZ et al.	230	N/A	N/A	SAS	Mild (≥50): 23.0%<br>Moderate (≥60): 7.0%	PTSD-SS	Screening positive (≥50): 27.4%

Study	Sample size	Screening tool: Depression	Prevalence; Cut-off score: %	Screening tool: Anxiety	Prevalence; Cut-off score: %	Screening tool: PTSD	Prevalence; Cut-off score: %
Huang Y et al.	2,250	CES-D	Screen positive: (≥28): 19.8%	GAD-7	Screen positive (≥9): 35.6%	N/A	N/A
Kounou et al.	62	PHQ-9	Mild (≥ 5): 51.6%<br>Moderate (≥ 10): 27.4%	GAD-7	Mild (≥ 5): 62.9%<br>Moderate (≥ 10): 37.1%	N/A	N/A
Lai et al.	1,257	PHQ-9	Mild (≥5): 50.4%<br>Moderate (≥10): 14.8%	GAD-7	Mild (≥5): 44.6%<br>Moderate (≥10): 12.3%	N/A	N/A
Lam et al.	932	PHQ-9	Screen positive (≥9): 24.4%	N/A	N/A	N/A	N/A
Li G et al.	4,369	PHQ-9	Screen positive (≥10): 14.2%	GAD-7	Screen positive (≥8): 25.2%	IES-R	Screen positive (≥33): 31.6%
Li J et al.	6,317	PHQ-9	Moderate (≥10): 21.3%	GAD-7	Moderate (≥10): 19.0%	N/A	N/A
Li RL et al.	66	N/A	N/A	HAMA	Screen positive (≥14): 77.3%	N/A	N/A
Li Z et al.	130	DASS-21	Screen positive (≥10): 26.2%	DASS-21	Screen positive (≥8): 77.7%	N/A	N/A
Liu S et al.	6,588	PHQ-9	Moderate (≥10): 57.6%	N/A	N/A	N/A	N/A
Liu X et al.	258	PHQ-4	Screen positive (≥3): 20.9%	PHQ-4	Screen positive (≥3): 19.8%	N/A	N/A
Liu Y et al.	1,315	PHQ-9	Moderate (≥10): 12.5%	GAD-7	Mild (≥5): 38.7%<br>Moderate (≥10): 10.7%	N/A	N/A
Liu Z et al.	4,679	SDS	Mild (≥50): 34.6%<br>Moderate (≥60): 19.7%	SAS	Mild (≥50): 16.0%<br>Moderate (≥60): 5.2%	N/A	N/A
Lu et al.	2,042	HAMD	Mild (≥7): 12.1%	HAMA	Mild (≥7): 25.5%	N/A	N/A
Lv et al.	8,028	N/A	N/A	GAD-7	Screen positive: 34.7%	N/A	N/A
Naser et al.	1,163	PHQ-9	Mild (≥5): 78.1%<br>Moderate (≥10): 44.8%	GAD-7	Mild (≥5): 70.8%<br>Moderate (≥10): 32.8%	N/A	N/A
Ni et al.	214	PHQ-2	Screen positive (≥3): 19.2%	GAD-2	Screen positive (≥3): 22.0%	N/A	N/A
Pouralizadeh et al.	441	PHQ-9	Mild (≥5): 71.0%<br>Moderate (≥10): 37.4%	GAD-7	Mild (≥5): 73.5%<br>Moderate (≥10): 38.8%	N/A	N/A
Qi et al.	400	SDS	Mild (≥50): 98.0%<br>Moderate (≥60): 26.0%	SAS	Mild (≥50): 31.0%<br>Moderate (≥60): 7.0%	N/A	N/A
Que et al.	2,285	PHQ-9	Mild (≥5): 55.6%<br>Moderate (≥10): 12.8%	GAD-7	Mild (≥5): 46.0%<br>Moderate (≥10): 11.6%	N/A	N/A
Rossi et al.	1,379	PHQ-9	Screen positive (≥15): 24.7%	GAD-7	Screening positive (≥15): 19.8%	GPS-PTSD	Screening positive: 49.5%
Salman et al.	398	PHQ-9	Mild (≥5): 63.8%<br>Moderate (≥10): 21.9%	GAD-7	Moderate (≥10): 21.4%	N/A	N/A
Shechter et al.	657	PHQ-2	Screen positive (≥3): 48.0%	GAD-2	Screen positive (≥3): 33.0%	N/A	N/A
Si et al.	863	DASS-21	Mild: 13.6%<br>Moderate: 6.0%	DASS-21	Mild: 13.9%<br>Moderate: 10.9%	IES-6	Screening positive: 40.2%

Study	Sample size	Screening tool: Depression	Prevalence; Cut-off score: %	Screening tool: Anxiety	Prevalence; Cut-off score: %	Screening tool: PTSD	Prevalence; Cut-off score: %
Song et al.	14,825	CES-D	Screen positive (≥16): 25.2%	N/A	N/A	PCL-5	Screening positive (≥33): 9.1%
Sun et al.	121	PHQ-9	Mild (≥5): 43.0%<br>Moderate (≥10): 13.2%	GAD-7	Mild (≥5): 58.7%<br>Moderate (≥10): 9.1%	N/A	N/A
Sung et al.	1,795	CES-D-10	Screen positive: (≥10): 45.5%	STAI-6	Mild (≥37): 100%<br>Moderate (≥44): 89.7%	N/A	N/A
Taghizadeh et al.	487	HADS	Mild (≥8): 48.3%<br>Moderate (≥11): 24.8%	HADS	Mild (≥8): 62.8%<br>Moderate (≥11): 34.3%	N/A	N/A
Tang et al.	44	SDS	Mild (≥50): 45.5%<br>Moderate (≥60): 18.2%	SAS	Mild (≥50): 31.8%<br>Moderate (≥60): 9.1%	N/A	N/A
Temsah et al.	582	N/A	N/A	GAD-7	Mild (≥5): 31.8%<br>Moderate (≥10): 11.0%	N/A	N/A
Tu et al.	100	PHQ-9	Mild (≥4): 46.0%<br>Moderate: 10.0%	GAD-7	Mild (≥4): 40.0%<br>Moderate: 7.0%	N/A	N/A
Wang Q et al.	342	HADS	Mild (≥8): 26.6%<br>Moderate (≥11): 10.5%	HADS	Mild (≥8): 28.1%<br>Moderate (≥11): 11.7%	N/A	N/A
Wang YX et al.	202	N/A	N/A	N/A	N/A	PCL-C	Screening positive (≥38): 16.8%
Weilenmann et al.	1,410	PHQ-9	Moderate (≥10): 20.7%	GAD-7	Moderate (≥10): 25.9%	N/A	N/A
Xiao et al.	958	HADS	Mild (≥8): 57.3%	HADS	Mild (≥8): 54.1%	N/A	N/A
Xie et al.	394	N/A	N/A	SAS	Mild (≥50): 35.0%<br>Moderate (≥60): 15.0%	N/A	N/A
Yao et al.	95	PHQ-9	Mild (≥5): 47.4%<br>Moderate (≥10): 20.0%	GAD-7	Mild (≥5): 50.5%<br>Moderate (≥14): 14.7%	N/A	N/A
Ye et al.	2,140	N/A	N/A	GAD-7	Mild (≥5): 43.6%<br>Moderate (≥10): 12.0%	N/A	N/A
Yin et al.	1,266	AFS	Mild (≥5): 27.5%<br>Moderate (≥11): 10.2%	AFS	Mild (≥7): 26.3%<br>Moderate (≥12): 11.7%	N/A	N/A
Zhang C et al.	1,563	PHQ-9	Mild (≥5): 50.6%<br>Moderate (≥10): 17.2%	GAD-7	Mild (≥5): 44.7%<br>Moderate (≥10): 12.9%	IES-R	Mild (≥9): 73.4%<br>Moderate (≥26): 37.4%
Zhang S et al.	304	PHQ-4	Screen positive (≥3): 20.6%	PHQ-4	Screen positive (≥3): 28.0%	N/A	N/A
Zhang WR et al.	927	PHQ-2	Screen positive (≥3): 12.2%	GAD-2	Screen positive (≥3): 13.0%	N/A	N/A

Study	Sample size	Screening tool: Depression	Prevalence; Cut-off score: %	Screening tool: Anxiety	Prevalence; Cut-off score: %	Screening tool: PTSD	Prevalence; Cut-off score: %
Zhao YJ et al.	209	PHQ-9	Mild (≥5): 34.4%<br>Moderate (≥10): 10.5%	GAD-7	Mild (≥5): 37.3%<br>Moderate (≥10): 12.0%	N/A	N/A
Zhao YP et al.	380	PHQ-9	Mild (≥5): 64.5%<br>Moderate (≥10): 21.1%	GAD-7	Mild (≥5): 51.3%<br>Moderate (≥10): 9.5%	N/A	N/A
Zhou et al.	210	PHQ-9	Mild (≥5): 72.0%<br>Moderate (≥10): 49.6%	GAD-7	Mild (≥5): 91.0%<br>Moderate (≥10): 76.6%	N/A	N/A
Zhu JR et al.	165	SDS	Screen positive (≥50): 44.2%	SAS	Screen positive (≥50): 20.0%	N/A	N/A
Zhu S et al.	858	PHQ-9	Screen positive (≥5): 19.2%	GAD-7	Screen positive (≥5): 18.0%	N/A	N/A

Abbreviation: AFS = Air Force Military Medical University Scale; BAI = Beck Anxiety Inventory; BDI = Beck Depression Inventory; CES-D = Center for Epidemiologic Studies Depression Scale; DASS = Depression Anxiety Stress Scales; GAD = Generalised Anxiety Disorder Assessment; GPS-PTSD = Global Psychotrauma Screen; HADS = Hospital Anxiety and Depression Scale; HAMA = Hamilton Anxiety Rating Scale; IES-R = Impact of Events Scale – Revised; PCL = PTSD Checklist; PCL-C = PTSD Checklist – Civilian Version; PHQ = Patient Health Questionnaire; PTSD-SS = Post-Traumatic Stress Disorder Self-Rating Scale; SAS = Zung's Self-Rating Anxiety Scale; SDS = Zung's Self-Rating Depression Scale; STAI = State Trait Anxiety Inventory

Screen positive = Proportion of participants scoring at or above the cut-off defined by the author to indicate clinically relevant depression, anxiety or PTSD
