# Supplementary material for: Prevalence of depression, anxiety and post-traumatic stress disorder in health care workers during the COVID-19 pandemic: A systematic review and meta-analysis
Source: PLoS One. 2021 Mar 10;16(3):e0246454. doi: 10.1371/journal.pone.0246454 (PMC7946321; doi:10.1371/journal.pone.0246454)
Supplement: S4 Appendix — (PDF) [file pone.0246454.s004.pdf]

S4 Appendix. Quality assessment and risk of study bias

Study	1. Is the source population representative of the population of interest?	2. Is the response rate adequate?	3. Is there little missing data?	4. Is the survey clinically sensible?	5. Is there any evidence for the reliability and validity of the survey instrument?
Amerio et al. (2020)					
Apisarnthanarak et al. (2020)					
Badahdah et al. (2020)					
Cao et al. (2020)					
Chatterjee et al. (2020)					
Chen J et al. (2020)					
Chen Y et al. (2020)					
Chew et al. (2020)					
Choudhury et al. (2020)					
Chung et al. (2020)					
Consolo et al. (2020)					
Du et al. (2020)					
Elbay et al. (2020)					
Evanoff et al. (2020)					
Gu et al. (2020)					
Guiroy et al. (2020)					
Guo et al. (2020)					
Gupta et al. (2020)					
Huang JZ et al. (2020)					
Huang Y et al. (2020)					
Kounou et al. (2020)					
Lai et al. (2020)					
Lam et al. (2020)					
Li G et al. (2020)					
Li J et al. (2020)					
Li RL et al. (2020)					
Li Z et al. (2020)					
Liu S et al. (2020)					
Liu X et al. (2020)					
Liu Y et al. (2020)					
Liu Z et al. (2020)					
Lu et al. (2020)					
Lv et al. (2020)					
Naser et al. (2020)					
Ni et al. (2020)					
Pouralizadeh et al. (2020)					
Qi et al. (2020)					
Que et al. (2020)					
Rossi et al. (2020)					
Salman et al. (2020)					
Shechter et al. (2020)					
Si et al. (2020)					
Song et al. (2020)					

Study	1. Is the source population representative of the population of interest?	2. Is the response rate adequate?	3. Is there little missing data?	4. Is the survey clinically sensible?	5. Is there any evidence for the reliability and validity of the survey instrument?
Sun et al. (2020)					
Sung et al. (2020)					
Taghizadeh et al. (2020)					
Tang et al. (2020)					
Temsah et al. (2020)					
Tu et al. (2020)					
Wang Q et al. (2020)					
Wang YX et al. (2020)					
Weilenmann et al. (2020)					
Xiao et al. (2020)					
Xie et al. (2020)					
Yao et al. (2020)					
Ye et al. (2020)					
Yin et al. (2020)					
Zhang C et al. (2020)					
Zhang S et al. (2020)					
Zhang WR et al. (2020)					
Zhao YJ et al. (2020)					
Zhao YP et al. (2020)					
Zhou et al. (2020)					
Zhu JR et al. (2020)					
Zhu S et al. (2020)					

Key

Definitely yes (low risk of bias)	
Probably yes	
Probably no	
Definitely no (high risk of bias)	
