# Supplementary material for: Prevalence of depression, anxiety and post-traumatic stress disorder in health care workers during the COVID-19 pandemic: A systematic review and meta-analysis
Source: PLoS One. 2021 Mar 10;16(3):e0246454. doi: 10.1371/journal.pone.0246454 (PMC7946321; doi:10.1371/journal.pone.0246454)

S6A Appendix. Assessment of publication bias by Begg's funnel plot and Egger's test for the studies reporting on the prevalence of anxiety in health care workers during the COVID-19 pandemic

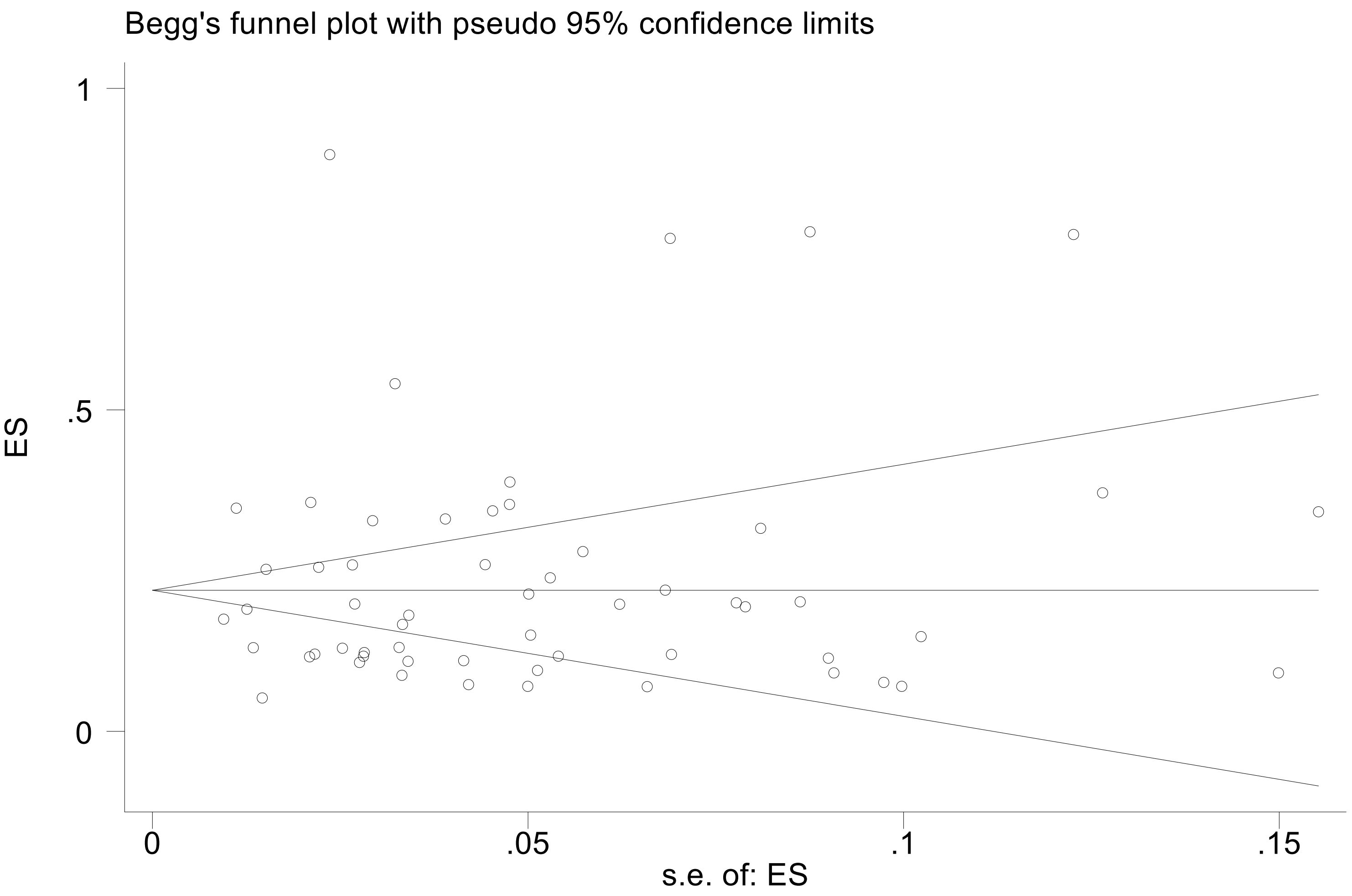

Egger's test: *p*=0.627

S6B Appendix. Meta-analysis and pooled estimate of mild anxiety among health care workers

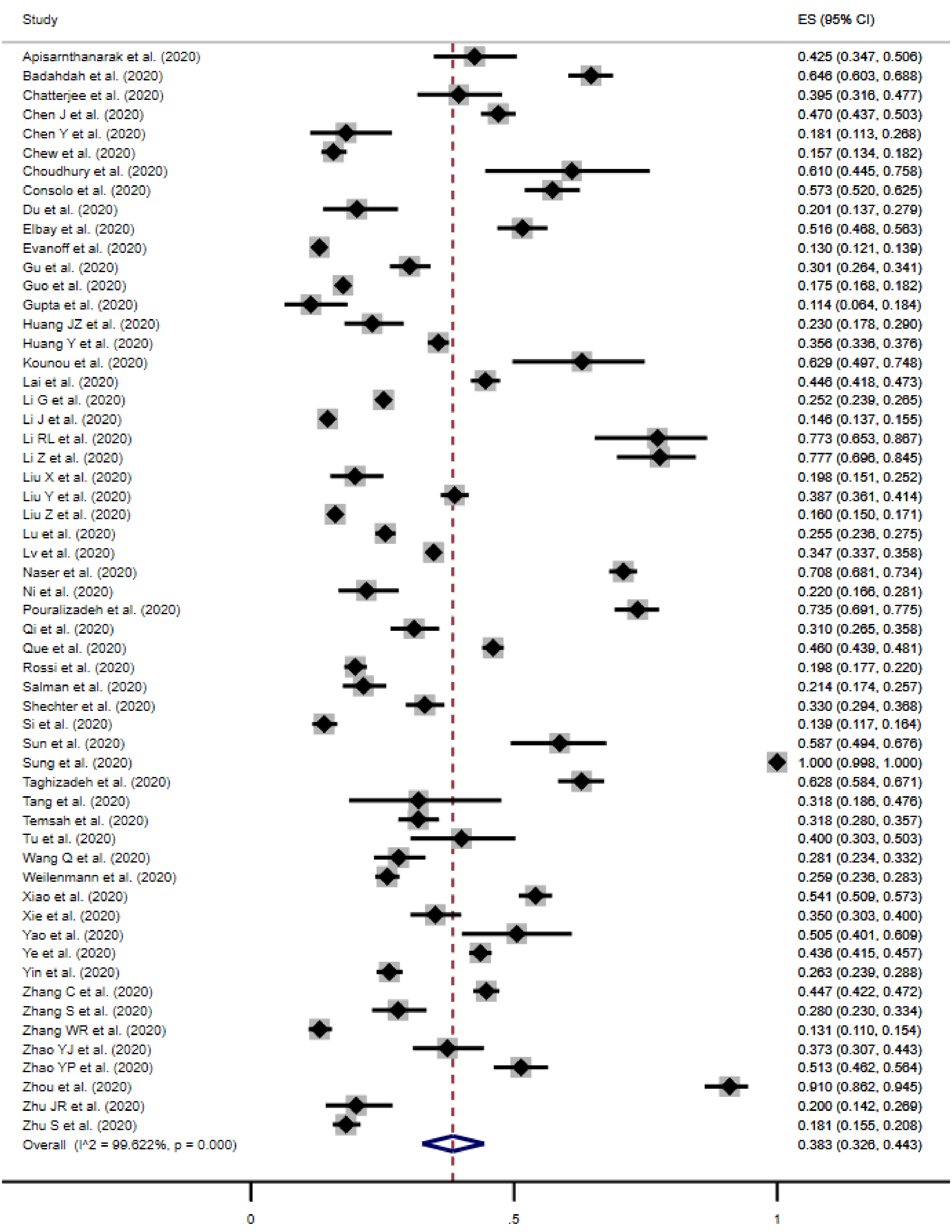

Supplement: S6 Appendix — (PDF) [file pone.0246454.s006.pdf]
